# Thermal Emission of Alkali Metal Ions from Al_30_-Pillared Montmorillonite Studied by Mass Spectrometric Method

**DOI:** 10.1155/2017/4984151

**Published:** 2017-10-08

**Authors:** V. B. Motalov, N. S. Karasev, N. L. Ovchinnikov, M. F. Butman

**Affiliations:** Ivanovo State University of Chemistry and Technology, Sheremetevsky Av. 7, Ivanovo 153000, Russia

## Abstract

The thermal emission of alkali metal ions from Al_30_-pillared montmorillonite in comparison with its natural form was studied by mass spectrometry in the temperature range 770–930 K. The measurements were carried out on a magnetic mass spectrometer MI-1201. For natural montmorillonite, the densities of the emission currents (*j*) decrease in the mass spectrum in the following sequence (T = 805 K, A/cm^2^): K^+^ (4.55 · 10^−14^), Cs^+^ (9.72 · 10^−15^), Rb^+^ (1.13 · 10^−15^), Na^+^ (1.75 · 10^−16^), Li^+^ (3.37 · 10^−17^). For Al_30_-pillared montmorillonite, thermionic emission undergoes temperature-time changes. In the low-temperature section of the investigated range (770–805 K), the value of *j* increases substantially for all ions in comparison with natural montmorillonite (T = 805 K, A/cm^2^): Cs^+^ (6.47 · 10^−13^), K^+^ (9.44 · 10^−14^), Na^+^ (3.34 · 10^−15^), Rb^+^ (1.77 · 10^−15^), and Li^+^ (4.59 · 10^−16^). A reversible anomaly is observed in the temperature range 805–832 K: with increasing temperature, the value of *j* of alkaline ions falls abruptly. This effect increases with increasing ionic radius of M^+^. After a long heating-up period, this anomaly disappears and the ln*j* − 1/*T* dependence acquires a classical linear form. The results are interpreted from the point of view of the dependence of the efficiency of thermionic emission on the phase transformations of pillars.

## 1. Introduction

Synthesis of functional materials (sorbents, molecular sieves, catalyst supports, solid electrolytes, etc.) based on layered aluminosilicates is a quickly developing research field [1]. A lot of work is carried out with montmorillonite (ММ) [2]. Its structure is characterized by a three-layered package (2 : 1), in which two tetrahedral sheets of silica (Т) sandwich a central octahedral sheet of alumina (О).

The silica-alumina layers possess a negative charge due to isomorphous substitution (e.g., Al^3+^ by Si^4+^ in T-layer and/or Al^3+^ by Mg^2+^ in O-layer). The neutrality of the mineral is provided by hydrated cations of alkali and alkali-earth metals presented in interlayer space.

Due to mobile alkali metal ions, MM is a natural solid electrolyte [3]. It is well known that solid electrolytes can emit conductivity ions on heating. For example, aluminosilicates with a skeleton structure such as zeolites were earlier investigated as alkali metal ion emitters characterized by stable thermal ion emission currents M^+^ (M is alkali metal) [4]. No similar study for MM, as far as we know, was performed. In consideration of the fact that the transportation of ions to a surface occurs by the internal channels of emitting substance [5], it is of interest to investigate not only natural but also modified ММ with extended distance between silicate layers, the so-called pillared MM. The latter can be produced by the intercalation of metal polyhydroxocomplexes into interlayer space of MM followed by calcining. As a result, nanocrystal periodical structures—pillars—arise, which are fixed by cross-linking with the silicate layers thus providing their significant separation. Consequently, pillared MM is characterized by large values of surface area and pore volume.

A crucial point of obtaining pillared MM is hydrolytic synthesis of intercalants—large-size multiple-charge metal polyhydroxocomplexes. This issue has most deeply been investigated for aluminium, whose hydrolysates in particular are [Аl_13_О_4_(ОН)_24_(Н_2_О)_12_]^7+^ ions (the so-called Keggin ions generally denoted by Al_13_). Hydrolysis of aluminium can be intensified under hydrothermal conditions provided by using an autoclave. In this way, stable «giant» ions with assumed formulae [Al_30_O_8_(OH)_56_(H_2_O)_24_]^18+^ (Al_30_)—adducts of the Keggin ions and Al(OH)_3_ molecules, the latter are bridges between Аl_13_ [6, 7]—can be synthesized in high concentration. The textural properties of the pillared MM intercalated by Al_30_ polycations were shown to be increased as compared with the one intercalated by Al_13_ [7, 8].

In this work, the Al_30_-pillared MM is investigated by a variant of the high temperature mass spectrometry allowing analyzing charged vapor species in thermal ion emission mode [9, 10]. Our aim was to determine the surface emission of alkali metal ions (Li^+^, Na^+^, K^+^, Rb^+^, and Cs^+^) as compared with the natural form of MM.

## 2. Experimental

### 2.1. Materials

MM was synthesized from bentonite of the Dash-Salakhly deposit, which is one of the best in Europe due to MM content [11], by conventional hydrosedimentation technique [12]. 20 grams of bentonite was dissolved in 1 liter of distilled water. In 24 hours, the top portion of the suspension was centrifugalized. The extracted fraction with 2-micrometer average particle size was dried at 60°С.

Pillaring solution containing giant Al_30_-polycations was obtained by receipt from [6] by hydrothermal processing (5-hour isothermal soaking at 127°С in an autoclave) of solution containing Al_13_-polycations. The latter was prepared by hydrolysis of aluminium chloride. In detail, 0.2 M solution of NaOH (Sigma Aldrich) was drop-by-drop added to 0.2 M solution of AlCl_3_·6H_2_O (Fluka) at room temperature and рН = 4.3–4.7 until a molar ratio [ОН^−^]/[Аl^3+^] = 2.4 has been attained followed by the solution aging at 60°С for 24 hours [13, 14]. Al_30_-pillared samples were obtained by calcining intercalated ones in oven at 350°С for 3 hours.

**Figure 1:**
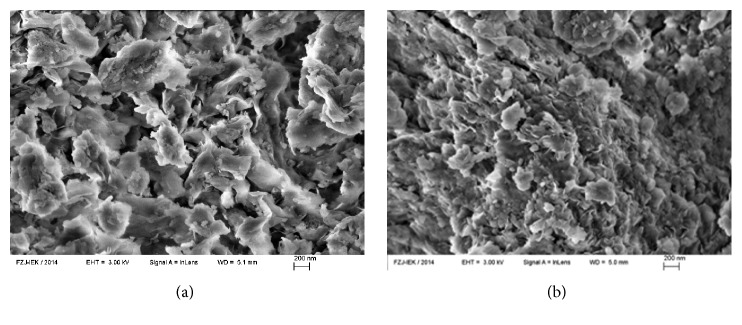
SEM image of natural MM (a) and Al_30_-pillared MM (b) samples.

The structural and textural properties of the obtained Al_30_-pillared MM are given in detail elsewhere [15]. For thermal ion emission studies, surface morphology of natural MM and Al_30_-pillared MM is of interest. The SEM images (scanning electron microscope Zeiss SUPRA 50VP, Germany) are shown in Figure 1. It can be seen from this figure that the original MM is composed of characteristic flaky particles of 100 to 1000 nanometers size, which «stick» to large-dimension aggregates. After modification with Al_30_-polycations, the sample of Al_30_-pillared MM exhibits more compact structure and a decrease in size of flaky particles. This fact is consistent with the larger value of specific surface area of Al_30_-pillared MM as compared to initial MM [15].

For thermal ion emission measurement, superfine powders of natural MM and Al_30_-pillared MM were formed in disks with 12 mm diameter and 1 mm depth using 0.2 GPa press.

### 2.2. Mass Spectrometric Technique

A single focusing sector type magnet mass spectrometer MI-1201 modified for high temperature experiments was used [16, 17]. Samples formed as disks were attached in molybdenum cylindrical holder at a depth of 2 mm from its surface. The holder with the disk was heated by tungsten-rhenium resistance furnace. To minimize temperature gradients, the heating assembly was surrounded by a set of tantalum radiation shields. The temperature was controlled by a tungsten-rhenium thermocouple calibrated in separate experiment with Ag. The accuracy of temperature measurement is estimated to be ±5 К. Ions emitted by a heated surface of the investigated samples in vacuum (10^−5^ Pa) were drawn by an electric field with a strength ~10^5^ V/m applied between the sample holder and a collimator (extracting electrode) attached at 7 mm distance from the disk surface. Ion beam passed through the collimator was focused by a system of electrostatic lenses and accelerated up to an energy 3 keV. Mass-to-charge separation of the ion beam occurred in a magnet field of electromagnet (angle 90°, radius of curvature 200 mm). A Hall probe measured magnetic field strength. Ion current registration system consisted of a secondary electron multiplier R595 (Hamamatsu, Japan) and a picoammeter 6485 (Keithley, USA).

## 3. Results and Discussion

**Figure 2:**
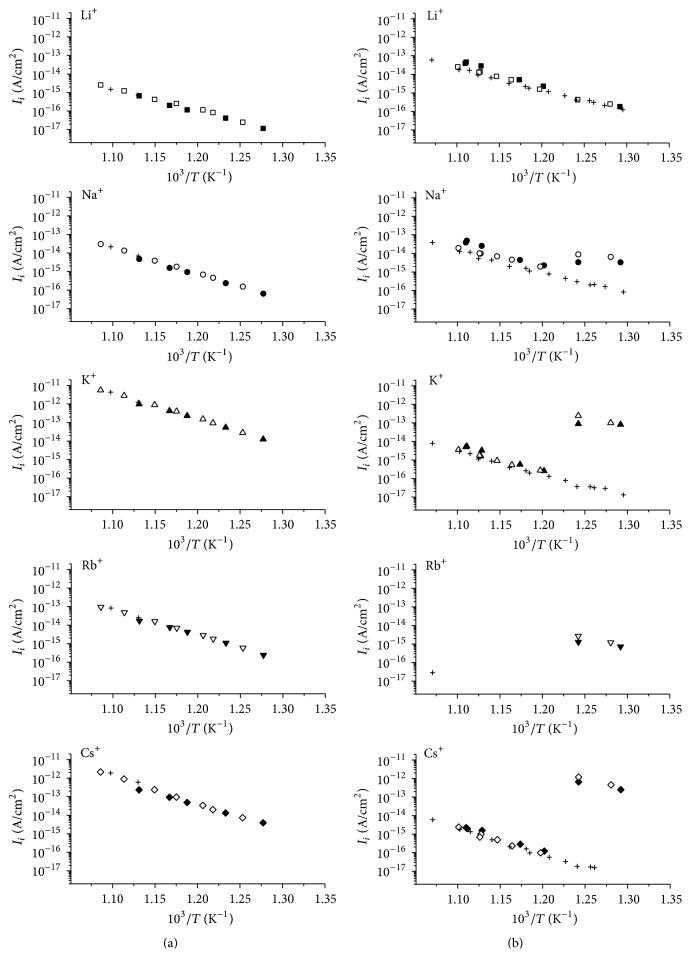
Temperature dependence of thermal emission ion currents measured for samples of natural MM (a) and Al_30_-pillared MM (b); open and solid symbols correspond to the first cooling and heating; crosses relate to repeated cooling and heating.

In mass spectra of thermal emission of both natural and Al_30_-pillared MM the ions of all alkali elements (Li^+^, Na^+^, K^+^, Rb^+^, and Cs^+^) were detected with various densities of emission current (*j*), whose temperature dependencies are shown in Figure 2 (the *j* values were calculated for a sum of isotope abundances).

The measurements were started from the highest temperature of the studied range and carried out in cooling and heating cycles. One can see in Figure 2 that, for both samples, dependencies ln⁡*I*_*i*_ = *f*(10^3^/*T*)  (*i* = Li^+^, Na^+^, K^+^, Rb^+^, Cs^+^) are reasonably reproduced on cooling and heating. Interestingly, on the dependencies for Al_30_-pillared MM in the first cooling and heating cycle, a discontinuity of monotonic course (a jump) of *j*-values is observed in the temperature range 805–832 K. At the same time, no similar effect appears for natural MM. It is noteworthy that the observed values of the ion current jump vary for different ions significantly. Namely, it is maximal for Cs^+^ (variation is characterized by a factor ~10^4^) and it decreases along a series Cs-(Rb)-K-Na-Li, that is to say, in accordance with cation size (it is not possible to estimate accurately the value of variation of the Rb^+^ ion current due to its low intensity at high temperatures of the investigated range). At the same time, the ion current jump effect is practically absent for Li^+^. After long heating, this anomaly disappears and the dependence ln⁡*j* − 1/*T* acquires classic linear form.

For comparison of emission phenomenon, in Table 1, the *j*-values for M^+^ ions at 805 K are given as an example; for Al_30_-pillared MM the data obtained in both initial stage, at which the emission anomaly was observed, and final stage of measurements are presented. It should be concluded from Figure 2 and Table 1 that, at low temperatures, the pillared sample demonstrates the higher emission as compared with natural MM. Its current density is several times higher for all alkali metal ions and it decreases in the raw Cs^+^, K^+^, Na^+^, Rb^+^, and Li^+^. In the high temperature region, as a result of stepwise decreasing, the emission of ions Na^+^ becomes comparable and for ions K^+^, Rb^+^, and Cs^+^ it decreases substantially as compared with the initial MM; *j*-values form a descending sequence Li^+^, Na^+^, K^+^, Cs^+^, and Rb^+^ thereby demonstrate the dependence on cation size.

Undoubtedly, among the data obtained in this work, the found effect of anomalous jump of emission current density in case of the pillared MM sample is the most interesting result. One can definitely state that this effect is connected with properties of pillars' ensemble. It is reasonable to assume that the rate of migration of emitted ions to emitter surface under applied electrical field depends significantly on structure peculiarities of inner surface of conductivity channels. Moreover, ion size can be an important factor influencing ion migration rates to surface [18]. In pillared MM, the area of inner surface of interlayer space is strongly conditioned by pillars themselves. Therefore, any structural modifications of pillars should apparently have an effect on effectiveness of thermal ion emission. It is very probable, in our opinion, that the temperature anomaly of emitting current is connected with chemical transformations of pillars and concurrent reactions with alkali metal ions. It is known that, in the course of aluminium hydroxide decomposition under normal air pressure, various structural types of aluminium oxide can be formed depending on thermal prehistory and the form of original substance [19, 20]; alkali metal admixtures can stabilize some kind of intermediate polymorphous modification. In our case the synthesized pillared MM can be considered as aluminosilicate matrix, in which interlayer space the boehmite-structured Al_30_-nanocrystals are regularly distributed.

It is also known that, firstly, the transformation *γ*-AlO(OH) → *γ*-А1_2_О_3_ in the temperature range 400–600°C is a classic example (including nanocrystals [21]) of topotactic reaction (boehmite-structure of *γ*-AlO(OH) remains [22]), and, secondly, in accordance with thermodynamic calculations [19] the formed *γ*-А1_2_О_3_ is nonstable in all temperature range of dehydration relative to an inverse process—hydration. Thus, heating of boehmite-like pillars should lead to formation of pillars with *γ*-Аl_2_О_3_ structure. The released molecules of water in this process can be used to form hydrated alkali metal ions M(H_2_O)_*n*_^+^. Their presence in interlayer space of MM allows providing reversibility of transformation of the *γ*-Аl_2_О_3_-like pillars into boehmite-like ones on cooling of ion emitter. Moreover, such interpretation allows explaining the anomaly effect disappearance in the last measurements of temperature cycle on cooling of the Al_30_-pillared MM sample. Vaporization of water from interlayer space during a long heating precludes the phase transformation of pillars with the *γ*-Аl_2_О_3_ structure into pillars with *γ*-AlO(OH) structure on decreasing temperature of ion emitter.

A physical reason of the anomaly itself is hypothetically a different surface morphology of two structural types of pillars. It is known that the phase transformation *γ*-AlO(OH) → *γ*-Аl_2_О_3_ leads to appearance of Al^3+^ cations on the crystal surface and structure modification connected with formation of a three-dimensional lattice with higher energy and stronger surface force field [23] instead of two-dimensional lattice. This circumstance can be a reason of increasing activation energy of thermal ion emission *E*_*a*_ and, correspondingly, decreasing thermal ion emission in case of *γ*-Аl_2_О_3_-pillars in comparison to *γ*-AlO(OH)-pillars. The values *E*_*a*_ calculated at harmonic mean temperature *T*_hm_ for original ММ and Al_30_-pillared MM are given in Table 2. One can see that the activation energies are higher for the assumed formed *γ*-Аl_2_О_3_-pillar ensemble than for the case of *γ*-AlO(OH)-pillars even with taking into account narrow temperature range and, hence, a large uncertainty of the measurement.

## 4. Conclusions

Thermal emission of alkali metal ions from natural MM is observed at temperatures higher than 770 K under used sensitivity of mass spectrometer 10^-17 ^A. The ratios of thermal ion current densities and activation energy of emission do not allow revealing key factors (ion radius, ionization energy of alkali metal, natural occurrence, etc.) determining physical principles of the phenomenon. Apparently, effectiveness of emission from MM depends strongly on surface concentration of ions and conditions of their migration from bulk to surface.

Thermal ion emission for the Al_30_-pillared MM undergoes temperature-time changes. At the initial stage of measurements in low-temperature area of the range studied (770–805 K) the *j*-value increases substantially for all ions in comparison with natural MM. A reversible anomaly is observed in the temperature range 805–832 K: with increasing temperature, the value of *j* of alkaline metal ions falls abruptly. This effect increases with increasing ionic radius of M^+^. After a long heating-up period, this anomaly disappears and the ln⁡*j* − 1/*T* dependence acquires a classical linear form. In this case, the values of *j* for ions with a small radius increase and for those with a large one decrease in comparison with the original montmorillonite.

Thus, in the example of investigation of thermal ion emission of Al_30_-pillared MM, it has been experimentally confirmed that this phenomenon is highly sensitive concerning defect and phase transformations of solid that were earlier found with ion single crystal [24–27]. In this work the anomalous temperature variations of thermal ion current from the surface of Al_30_-pillared MM are connected, in authors' opinion, with polymorphous transformations of pillars-nanoparticles ensemble. Validation of this hypothesis requires further experiments with other types of pillared materials.

## Figures and Tables

**Table 1:** Thermal emission current density of M^+^ ions (*T* = 805 K).

Sample	*j*, A/cm^2^				
	Li^+^	Na^+^	K^+^	Rb^+^	Cs^+^
Natural MM	3.37 · 10^−17^	1.75 · 10^−16^	4.55 · 10^−14^	1.13 · 10^−15^	9.72 · 10^−15^
Al_30_-pillared MM (initial stage)	4.59 · 10^−16^	3.34 · 10^−15^	9.44 · 10^−14^	1.77 · 10^−15^	6.47 · 10^−13^
Al_30_-pillared MM (final stage)	4.47 · 10^−16^	2.97 · 10^−16^	3.98 · 10^−17^	Under sensitivity limit	1.80 · 10^−17^

**Table 2:** Activation energies of thermal ion emission.

Sample	Δ*T*, K	*T*_hm_, K	*E*_*a*_(*T*_hm_), kJ mol^–1^				
			Li^+^	Na^+^	K^+^	Rb^+^	Cs^+^
Al_30_-pillared MM (initial stage)	774–805	791	143 ± 13	53 ± 108	100 ± 95	145 ± 76	196 ± 64
Al_30_-pillared MM (final stage)	772–934	841	227 ± 5	227 ± 6	240 ± 7	—	271 ± 8
Natural MM	783–921	852	232 ± 7	271 ± 6	270 ± 6	264 ± 6	290 ± 10
